# Functional significance of vertical free moment for generation of human bipedal walking

**DOI:** 10.1038/s41598-023-34153-4

**Published:** 2023-04-27

**Authors:** Takuo Negishi, Naomichi Ogihara

**Affiliations:** Department of Biological Sciences, Graduate School of Science, The University of Tokyo, 7-3-1, Hongo, Bunkyo-ku, Tokyo, 113-0033 Japan

**Keywords:** Biomechanics, Biological anthropology

## Abstract

In human bipedal walking, the plantar surface of the foot is in contact with the floor surface, so that a vertical free moment (VFM), a torque about a vertical axis acting at the centre-of-pressure due to friction between the foot and the ground, is generated and applied to the foot. The present study investigated the functional significance of the VFM in the mechanics and evolution of human bipedal walking by analysing kinematics and kinetics of human walking when the VFM is selectively eliminated using point-contact shoes. When the VFM was selectively eliminated during walking, the thorax and pelvis axially rotated in-phase, as opposed to normal out-of-phase rotation. The amplitudes of the axial rotation also significantly increased, indicating that the VFM greatly contributes to stable and efficient bipedal walking. However, such changes in the trunk movement occurred only when arm swing was restricted, suggesting that the VFM is critical only when arm swing is restrained. Therefore, the human plantigrade foot capable of generating large VFM is possibly adaptive for bipedal walking with carrying food, corroborating with the so-called provisioning hypothesis that food carrying in the early hominins is a selective pressure for the evolution of human bipedalism.

## Introduction

In human bipedal walking, the plantar surface of the foot is in surface contact with the floor, so that the ground reaction forces (GRFs) are applied to the foot in a distributed manner, generating a vertical free moment (VFM), i.e., the torque about a vertical axis acting at the centre of pressure due to friction between the foot and the ground^1–8^. Numerous studies have been published elucidating GRF during human walking to explain the biomechanics and motor control of human bipedal locomotion^9,10^. However, the VFM was often neglected in these studies, and studies focusing on the VFM have been limited until recently^1–8^, despite its potential importance for regulation of rotational stability of bipedal walking in humans. These previous studies have identified that (1) the VFM is acting in the direction of external and internal rotations during the first and second half of the stance phase during walking^1,3–7^; (2) the magnitude of the VFM is greater in the second half of the stance phase^1,3–7^; (3) the VFM is strongly affected by arm swing and the peak magnitudes of the VFM increase when arm swing is restrained, resulting in higher energy expenditure in locomotion^2,3,5,8^; and (4) the VFM is also affected by gait speed, and the peak magnitudes of the external and internal rotations decrease and increase, respectively, as gait speed increases^1,7^. However, to date, no studies have attempted to identify the true biomechanical impact of the VFM on bipedal walking in humans by “knocking out” or selectively eliminating VFM that is applied to the foot while walking. Therefore, it remains largely unclear how and to what extent does the VFM contribute to the generation of stable and efficient human bipedal walking.

Understanding the contribution of the VFM in the regulation of the body’s rotational movement around the vertical axis during human walking is also important in the context of the evolution of human bipedalism. The ultimate and proximate causes for the evolution of habitual bipedalism remain a key question in palaeoanthropology. A number of hypotheses have been proposed for the positive selective pressure for human habitual bipedal locomotion, including the feeding strategy^11^, predator avoidance^12^, thermoregulation^13^, locomotor efficiency^14^, and foraging in a wetlands habitat^15^ or on flexible branches^16^. However, why the adoption of upright bipedal locomotion can lead to improved reproductive success of our ancestors in the course of human evolution still remains largely obscure. One of the possible selective advantages of bipedal locomotion is that it releases the arms so that they can be used to carry things, such as food items and infants. However, carrying food items or infants certainly restricts arm swing during walking, possibly requiring generation of larger magnitude of VFM. Under such circumstances, the derived plantigrade foot of humans^17–19^ may facilitate generation of larger VFM because of the increased moment arms of the horizontal GRFs with respect to the centre-of-pressure (COP). Therefore, it could be anticipated that the human plantigrade foot might have evolved as an adaptation to facilitate generation of rotationally stable bipedal walking while carrying things, such as food items and infants, by counterbalancing the vertical moment of the body.

**Figure 1:**
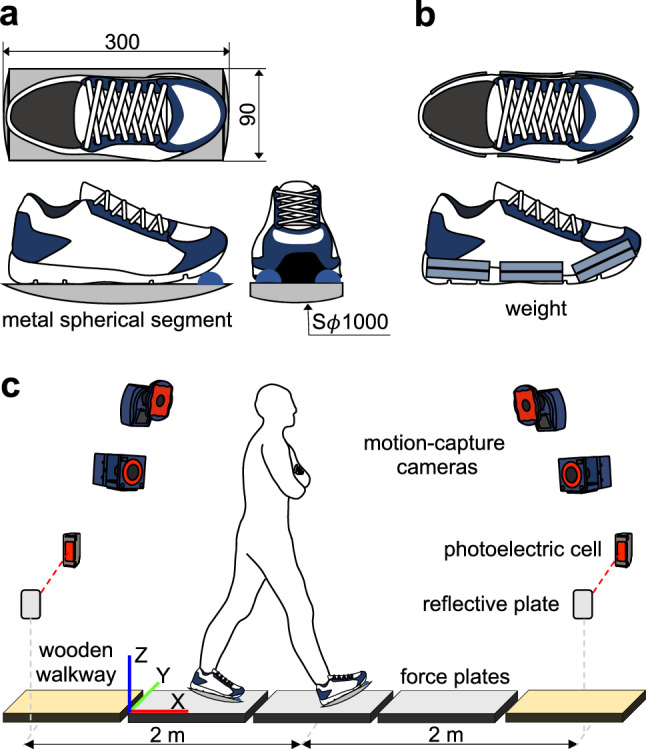
Point-contact (PC) shoe (**a**), normal (NL) shoe (**b**), and experimental setup (**c**).

The present study kinematically and kinetically investigated the functional significance of VFM in human bipedal walking by analysing human walking when the VFM is selectively eliminated. For this purpose, a pair of ‘point-contact (PC) shoes’ with a metal spherical segment (aluminium, Sφ1000 mm, 550 g) attached to each sole of an ordinary athletic shoe was fabricated (Fig. 1a), and the body kinematics and kinetics during bipedal walking with the PC shoes were compared with those with a pair of ‘normal (NL) shoes’ with a similar weight to that of the metal spherical segment attached (Fig. 1b). Walking with the normal shoes with the attached weight had no effect on the kinematics and kinetics of walking (Supplementary Fig. S1–3). As the VFM is known to be affected by arm swing^1,3,5,8^, arm swing was restrained by asking the participants to fold their arms while conducting the walking experiment. Therefore, the present study primarily compared bipedal walking using the PC and NL shoes without arm swing (PCwoAS and NLwoAS, respectively) to test our null hypothesis that the selective elimination would not affect the kinematics and kinetics of human bipedal walking. In addition, to investigate the possible contribution of arm swing when the VFM is selectively eliminated, we also investigated bipedal walking with the PC shoes and arm swing (PCwAS) for comparisons, and tested whether kinematics and kinetics of bipedal walking in the above three conditions are equal to one another.

## Results

### Spatiotemporal parameters

The mean gait cycle duration (1.24 ± 0.07 s, 1.23 ± 0.07 s, and 1.25 ± 0.07 s), stride length (1.36 ± 0.07 m, 1.37 ± 0.07 m, and 1.37 ± 0.07 m), and speed (1.10 ± 0.02 m/s, 1.10 ± 0.02 m/s, and 1.10 ± 0.02 m/s) were essentially identical among the three conditions, PCwoAS, NLwoAS, and PCwAS, respectively.

### Ground reaction forces

**Figure 2:**
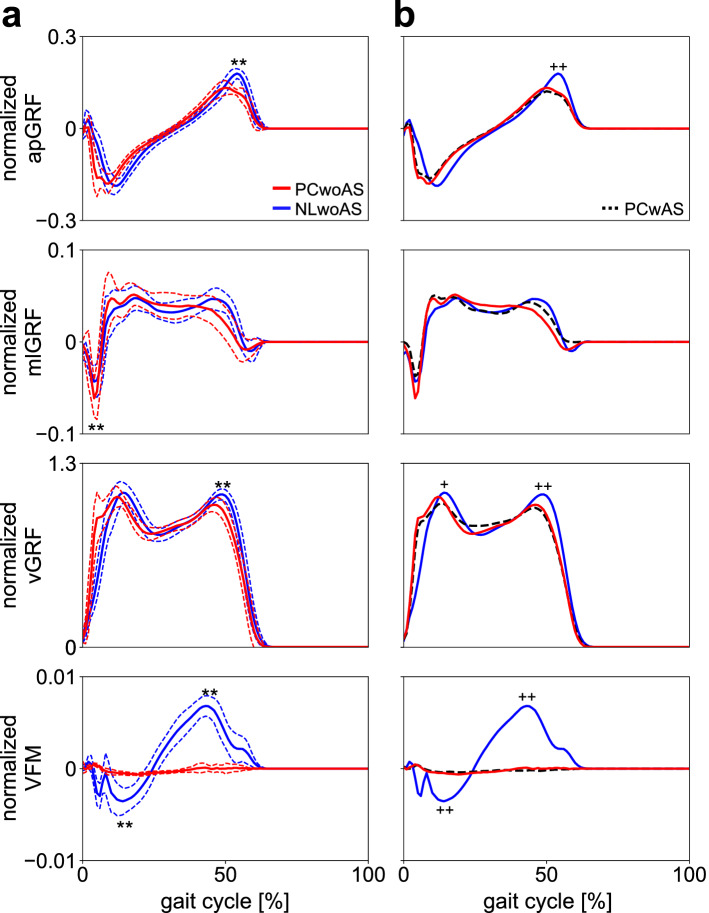
Mean normalised GRF and VFM profiles during walking with NL and PC shoes when arm swing was restrained (NLwoAS and PCwoAS, respectively) (**a**). Mean normalised GRF and VFM profiles were also compared with those using the PC shoes when the arm swing was not restrained (PCwAS) (**b**). Blue solid line = NLwoAS, Red solid line = PCwoAS. Corresponding dashed lines represent standard deviations. Black dashed line = PCwAS. Asterisks indicate statistical differences of the maximum or minimum values between NLwoAS and PCwoAS (*: *p* < 0.05. **: *p* < 0.01). Pluses indicate statistical differences between NLwoAS and PCwAS (+: *p* < 0.05. ++: *p* < 0.01).

Three components of the GRF profiles exhibiting typical waveforms of bipedal walking were very similar for all three conditions. Although there were statistically significant differences in the peak magnitudes of the propulsive, lateral, and the first and second vertical GRFs among the three conditions (Fig. 2), these differences remain small in absolute values. The VFM profile of NLwoAS also exhibited a typical waveform of the VFM during human walking. However, during bipedal walking with the PC shoes (PCwoAS and PCwAS), the VFM profiles were nearly zero throughout the stance phase. Therefore, the use of the PC shoes successfully eliminated the VFM applied to the foot during walking without affecting the shape and magnitude of the three components of the GRF vector (Fig. 2).

### Segmental and joint angles

**Figure 3:**
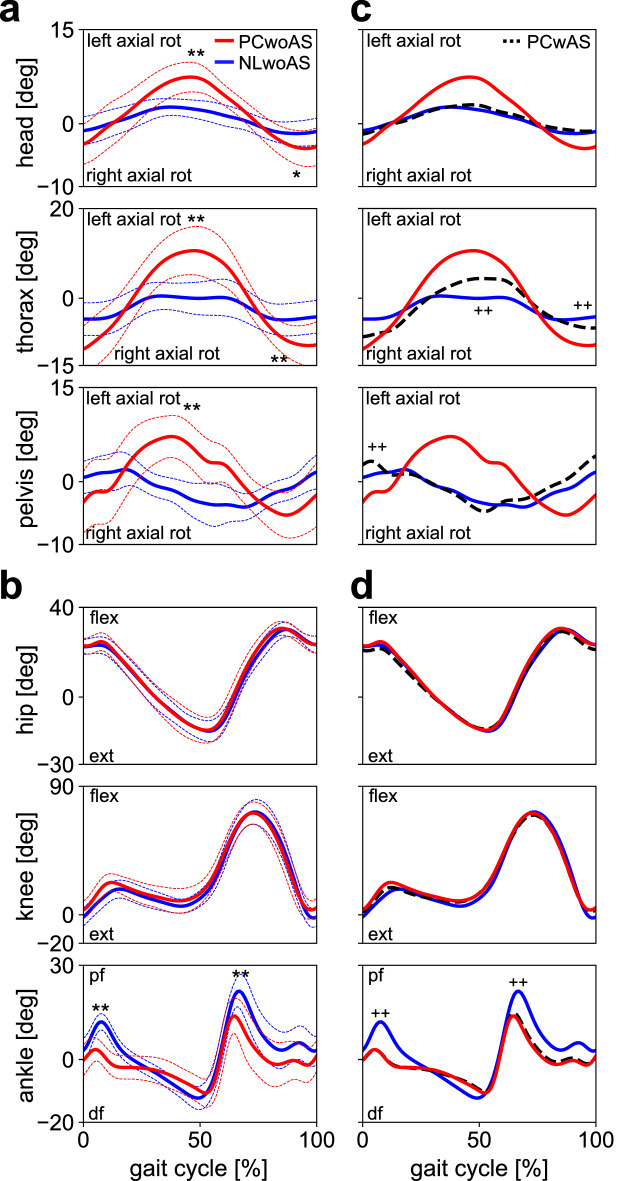
Mean trunk segment (**a**) and joint angle (**b**) profiles during walking with NL and PC shoes when the arm swing was restrained (NLwoAS and PCwoAS, respectively). Mean segment and joint angle profiles were also compared with those with PC shoes when the arm swing was not restrained (PCwAS) (**c**, **d**). Blue solid line = NLwoAS, Red solid line = PCwoAS. Corresponding dashed lines represent standard deviations. Black dashed line = PCwAS. Asterisks indicate statistical differences of the maximum or minimum values between NLwoAS and PCwoAS (*: *p* < 0.05. **: *p* < 0.01). Pluses indicate statistical differences between NLwoAS and PCwAS (+: *p* < 0.05. ++: *p* < 0.01).

The ranges of axial rotation of the head, thorax, and pelvis segments relative to the global coordinate system were significantly larger in PCwoAS than in NLwoAS (head max: 7.5 ± 2.4 deg vs. 2.9 ± 1.4 deg [p = 0.001], head min: − 4.2 ± 3.0 deg vs. − 1.9 ± 2.0 deg [p = 0.041], thorax max: 11.1 ± 5.5 deg vs. 1.5 ± 3.5 deg [p < 0.001], thorax min: − 12.5 ± 5.0 deg vs. − 5.6 ± 3.7 deg [p = 0.006], and pelvis max: 7.6 ± 3.9 deg vs. 3.2 ± 2.3 deg [p = 0.004] and pelvis min: − 6.1 ± 3.7 deg vs. − 5.0 ± 1.6 deg [n.s.] for PCwoAS and NLwoAS, respectively) (Fig. 3a). In addition, the thorax and pelvis segments rotated in-phase in PCwoAS, but the thorax and pelvis rotated out-of-phase in NLwoAS (Fig. 3a). The hip and knee joint angle profiles were identical between the two conditions, but the peak plantarflexion of the ankle joint in the early and late stance phases was slightly but significantly smaller in PCwoAS than in NLwoAS (Fig. 3b).

If PCwoAS and NLwoAS were compared with PCwAS, it was found that the segmental angle profiles of the head, thorax, and pelvis segments in PCwAS were more similar to those of NLwoAS than those of PCwoAS (Fig. 3c). Particularly, the thorax and pelvis rotated in-phase in PCwoAS, but they rotated out-of-phase when arm swing was not restrained (in PCwAS) as in NLwoAS. The hip, knee and ankle joint angle profiles during bipedal walking with the PC shoes did not change by the presence or absence of arm swing (Fig. 3d).

### Whole-body angular momentum

**Figure 4:**
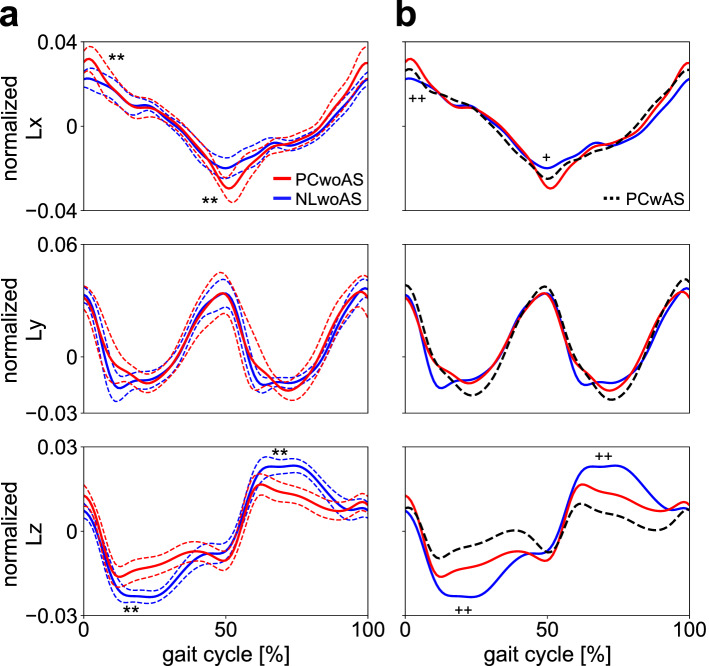
Mean normalised WBAM profiles of the body COM during walking with the NL and PC shoes when the arm swing was restrained (NLwoAS and PCwoAS, respectively) (**a**). Mean profiles were also compared with those with the PC shoes when the arm swing was not restrained (PCwAS) (**b**). Blue solid line = NLwoAS, Red solid line = PCwoAS. Corresponding dashed lines represent standard deviations. Black dashed line = PCwAS. Asterisks indicate statistical differences of the maximum or minimum values between NLwoAS and PCwoAS (*: *p* < 0.05. **: *p* < 0.01). Pluses indicate statistical differences between NLwoAS and PCwAS (+: *p* < 0.05. ++: *p* < 0.01).

The magnitude of the whole-body angular momentum (WBAM) around the anterior and vertical axes (Lx and Lz) during walking was significantly larger (Lx max: 0.033 ± 0.006 vs. 0.024 ± 0.004 [p < 0.001], and Lx min: − 0.031 ± 0.005 vs. − 0.022 ± 0.004 [p = 0.002] in PCwoAS and NLwoAS, respectively) and smaller (Lz max: 0.017 ± 0.004 vs. 0.024 ± 0.003 [p < 0.001], and Lz min − 0.017 ± 0.004 vs. − 0.024 ± 0.002 [p < 0.001] for PCwoAS and NLwoAS, respectively) in PCwoAS than in NLwoAS (Fig. 4a). In PCwAS, the WBAM around the vertical axis (Lz) was further lessened by arm swing (Lz max: 0.010 ± 0.004 vs. 0.017 ± 0.004 [p < 0.001], and Lz min − 0.010 ± 0.004 vs. − 0.017 ± 0.004 [p < 0.001] for PCwAS and PCwoAS, respectively) (Fig. 4b).

### Vertical external moment due to GRF and VFM

**Figure 5:**
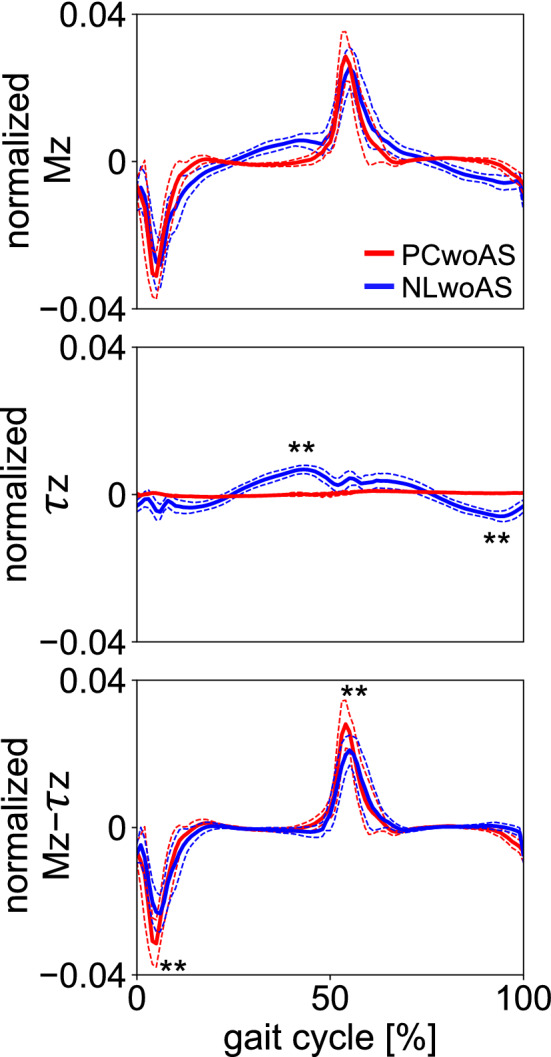
Mean normalised vertical external moment profiles during walking with NL and PC shoes when the arm swing was restrained (NLwoAS and PCwoAS, respectively). Normalised net moment (M_z_), moment due to VFM (τ_z_ = τ_zL_ + τ_zR_), and moment due to GRF (M_z_−τ_z_) are presented. Corresponding dashed lines represent standard deviations. Asterisks indicate statistical differences of the maximum or minimum values between NLwoAS and PCwoAS (*: *p* < 0.05. **: *p* < 0.01).

The net external moment (M_z_) was the largest in the magnitude during double-support phase in both PCwoAS and NLwoAS (Fig. 5), and the main component was the moment profile due to GRF (M_z_–τ_z_). However, the external moment due to GRF was nearly zero during the single-support phase. The amplitude of the VFM (τ_z_) was much smaller than the moment due to GRF, but the VFM was generated during the single-support phase when the external moment due to GRF was nearly zero.

## Discussion

Our results showed that when the VFM was selectively eliminated during human bipedal walking by wearing the PC shoes, the trunk and pelvis segments rotated in-phase, but the thorax and pelvis rotated out-of-phase if the participants walked with the NL shoes (Fig. 3a). The range of in-phase motion of the head, thorax, and pelvis segments relative to the global coordinate system were significantly larger in walking with the PC shoes than with the NL shoes (Fig. 3a). This indicates that the trunk motion was less stable if the VFM was eliminated. This is consistent with previous studies reporting that the out-of-phase rotations of the trunk and pelvis and arms and legs improve rotational stability of walking gait^20–22^ in emphasised arm swing.

Nevertheless, the magnitude of the WBAM around the vertical axis (Lz) during walking was significantly smaller while walking with the PC shoes than while walking with the NL shoes (Fig. 4). This is attributed to the fact that the vertical WBAM should be intentionally minimised to walk with the PC shoes because the vertical WBAM cannot be counteracted by the VFM. In walking with the PC shoes, the present study observed that the vertical WBAM was reduced by a decrease in vertical angular momenta of the thigh segments, the main component of the vertical WBAM (Supplementary Fig. S4). This was done by axially rotating the pelvis segment in-phase with the thorax segments (Fig. 3a), as this reduces the anteroposterior velocities of the thigh centres-of-mass (COMs). It further explains why the thorax and pelvis axially rotates in-phase in walking with the PC shoes, different from walking with the NL shoes. The cancellation of the WBAM was additionally achieved by increasing the axial rotation and angular momentum of the thorax (Fig. 3a) as well as the head and arm moving with the thorax (Fig. 3a; Supplementary Fig. S4), resulting in further reduction in the WBAM around the vertical axis in walking with the PC shoes. However, such in-phase axial rotation of the pelvis with respect to the thorax and the large axial rotation of the thorax were not observed in walking with the NL shoes, despite the smaller vertical WBAM. This is attributed to the fact that generating such large in-phase axial rotations of the thorax and pelvis is energetically more costly than generating out-of-phase axial rotations as in normal human walking. In walking with the PC shoes (with in-phase axial rotations of the thorax and pelvis), resonant torsional oscillation between the thorax and pelvis segments due to muscle-springs in the human trunk^23^ as well as the resultant passive movements of the thigh segments had to be intentionally suppressed and eliminated by active muscle control of the trunk segments, resulting in increased cost of locomotion^2,3,5^. However, if a sufficient frictional moment can be generated between the plantar surface of the foot and the ground, the vertical WBAM should be passively compensated and counteracted by the VFM, perhaps resulting in improved locomotor efficiency of bipedal walking. Therefore, the present study suggests that the capacity of the human foot to generate VFM is essential for generation of stable as well as efficient bipedal locomotion, despite the magnitude of VFM being much smaller than the vertical external moment due to the two components of the horizontal GRFs (Fig. 5).

The present study also observed that even when the VFM was eliminated during walking with the PC shoes, if the arm swing was not restrained, the thorax and pelvis could rotate out-of-phase and the ranges of rotation were much reduced as observed in walking with the NL shoes (Fig. 3c), even though this did not affect the kinematics of lower limb angles (Fig. 3d). This result suggests that active arm swinging out-of-phase with the legs could induce generation of the out-of-phase rotation of the two segments to reduce the vertical WBAM even when no VFM is applied to the body during walking (Fig. 4). Therefore, the ability to generate the VFM is particularly critical for generating stable and efficient bipedal walking, especially when the arm swing is restrained, for instance, in case of object carrying.

The human foot uniquely possesses an enlarged, robust calcaneus, the tuberosity of which points inferoposteriorly, allowing prominent heel strike during walking^17–19^. In addition, the human foot is unique in having comparatively short toes than non-human primates^24,25^. Therefore, the area of the plantar surface of the human foot is comparatively much larger in the anteroposterior direction than those of the other non-human primates. Furthermore, the human foot lost opposable hallucis^26,27^, indicating that the plantar surface of the foot (thenar eminence) is mediolaterally wider in humans compared to other non-human primates due to the presence of the first ray next to the second ray in parallel (the opposable hallux in the chimpanzee does not contribute much to the generation of large VFM because the mobile hallux can easily move due to forces applied to it). The large contact area between the plantar surface of the foot and the ground may provide a larger moment arm of the horizontal GRFs with respect to the COP. Nevertheless, the human foot uniquely possesses plantar arch that reduces the surface contact area, but what is important here is the large contact area distant from the COP. Therefore, these unique features of the human foot possibly contributed to the generation of a large VFM. In fact, plantigrade feet were reportedly capable of producing larger VFM to the ground than digitigrade feet in human participants resisting against vertically oriented external torsional moment applied to their body^28^. In addition, the human foot is known to possess a unique capacity to generate a VFM during axial loading^29,30^ due to the kinematic coupling of the calcaneus and tibia, the so-called tibio-calcaneal coupling^31–33^. Owing to the innate morphology of the human foot, axial loading of the human foot resulted in eversion of the calcaneus and internal rotation of the talus and tibia^29,30,34^, which in turn resulted in generation of ground reaction moment around the vertical axis of the ground (VFM)^30,35^. However, the coupling motion of the calcaneus and tibia and the resultant VFM were shown to be much smaller in the chimpanzee foot^29,30^. Therefore, the human foot might have evolved to produce a large VFM, possibly to facilitate generation of stable and efficient bipedal walking particularly when arm swing is restrained. Recent studies have suggested that evolutionary changes in the compliance of the foot have been the key for the evolution of human bipedal walking and running^36–38^, but selection for the capacity of the foot to produce large VFM may also have been a critical factor for the evolution of the derived features of the human foot anatomy.

Currently, one of few hypotheses concerning the origin of the evolutionary transition to bipedalism accompanied by substantial paleontological contexts is the so-called provisioning hypothesis^39–41^. This hypothesis has become more accepted particularly after the discovery of *Ardipithecus* demonstrating only a low degree of sexual dimorphism^42^. In this hypothesis, positive selective pressure was considered to be imposed on pair-bonded male individuals, provisioning dependent females, and offspring by walking on two legs and carrying food items in the freed arms. Such provisioning likely leads to increase in their survival fitness and reproductive success. In addition, walking on two legs allows female individuals to carry infants who were probably born relatively immature in early hominins^43,44^, which could further contribute to increased reproductive success. If the arms are used for carrying food items or an infant, the arm motions should be very much restricted, and the arms cannot be used for regulation of the WBAM during walking. Therefore, the present results show that the ability to generate VFM is critical for generating stable and efficient bipedal walking when arm swing is restrained, thereby supporting the provisioning hypothesis. It should be noted that *Ardipithecus* had an opposable hallux, indicating that the capacity to generate VFM was probably more restricted as it is in chimpanzees^29,30^. Therefore, the foot of early hominins was not as adapted to habitual bipedal walking as that of modern humans. However, the morphology of the foot in early hominins should have been gradually derived to be more human-like up to the time of *Australopithecus* and early *Homo* through the course of human evolution possibly due to continuous selective pressure on male provisioning and bipedal walking. Therefore, the fact that the derived characteristics of human foot facilitated production of larger VFM^28,30^ further advocates that food provisioning of dependent females and offspring by males and infant carrying by females in early hominins are selective pressures for the evolution of human bipedalism as well as the corresponding anatomy of the human foot.

The present study has several limitations. First, this study did not attempt to directly quantify the difference in the energetic cost of locomotion during bipedal walking with the PC and NL shoes. A future study should directly confirm whether the in-phase and larger axial rotation of the thorax and pelvis observed when the VFM was selectively eliminated during walking actually reduces the energetic efficiency of locomotion by using expiratory gas analysis. Second, the present study analysed bipedal walking when arm swing was restrained by asking the participants to fold their arms, but not asking them to carry actual food items or a mannequin of an infant, because what to carry (size, shape, and weight) was difficult to choose, and normalisation of the object size, shape, and weight could also be troublesome. However, a future study should also confirm whether bipedal walking when the VFM was selectively eliminated is actually deteriorated in terms of stability and energetic efficiency by carrying objects, such as food items and an infant mannequin. Third, only adult male individuals were recruited for the experiment. Although absolute differences of bipedal walking between sexes are small, the current findings should also be confirmed by female participants in the future.

## Methods

### Point contact shoes

A pair of point-contact (PC) shoes with a metal spherical segment (aluminium, Sφ1000 mm, 550 g) bonded to each sole of an ordinary athletic shoe (JOG100-2; Asics, Kobe, Japan) was fabricated to eliminate VFM during walking (Fig. 1a). To fill the gap between the dorsal surface of the metal sphere and the plantar surface of the toe of the shoe, a rubber hemisphere was placed. A pair of the same athletic shoes attached with weight (lead plates) of the same mass as the metal sphere was used for comparisons (‘NL shoes’, Fig. 1b). We confirmed that adding the weight of 550 g on each shoe had virtually no effect on the kinematics and kinetics of walking (Supplementary Fig. S1–3). It was also confirmed that the VFM profiles of barefoot and shoes walking were essentially identical to each other due to the principle of action and reaction.

### Participants

Ten adult male individuals without any history of orthopaedic or neuromuscular impairments (mean [± standard deviation] age, 26.7 [± 3.5] years; height, 1.70 [± 0.04] m; weight, 61.5 [± 5.0] kg; and COM height in quiet standing, 0.98 ± 0.02 m) participated in the study. Informed consent was obtained from each participant. This study was reviewed and approved by the Office for Life Science Research Ethics and Safety at the University of Tokyo. All methods were carried out following the relevant guidelines and regulations.

### Experimental procedure

The participants walked across three 600 mm × 400 mm force plates (EFP-S-1.5KNSA13; Kyowa Dengyo, Tokyo, Japan) set in a wooden walkway (8.2-m long) with the NL and PC shoes of appropriate size (Fig. 1c). Body kinematics were recorded using a motion capture system consisting of 10 cameras (MAC3D; Motion Analysis Corporation, Santa Rosa, CA). A total of 29 reflective markers were attached to the body based on the modified Helen Hayes marker set (Supplementary Fig. S5)^45^, and the positions of the markers were captured at 100 Hz. The GRF signals were simultaneously recorded at 200 Hz using a universal recorder (EDX-100A; Kyowa Dengyo, Tokyo, Japan). The participants were asked to walk with restricted arm swing by folding their arms. In the experiment, the participants firstly walked with the NL shoes and, then, with PC shoes (NLwoAS and PCwoAS, respectively). To adapt to the walking with the PC shoes, the participants were allowed to initially walk back and forth along the walkway for 5 min and, then, on a 1.8-m long treadmill (DLF-55E, Ohtake Root Kogyo, Ichinoseki, Japan) set at 4 km/h for 10 min as a practice session. We also asked the participants to walk with the PCwAS for comparisons. We did not randomise the order of conditions to eliminate possible after-effects in walking patterns following adaptation to the PC shoes. The participants were instructed to step on the three force plates with their left, right, and left feet, respectively. Two pairs of photoelectric cells were placed 4 m apart across the force plates to instantaneously measure walking speed of each trial. Trials with walking speed within 5% of the target speed (1.1 m/s) were selected as successful trials, and five successful trials were recorded for each condition.

### Data analysis

The gait cycle duration, stride length (horizontal distance travelled in a gait cycle), and speed were calculated using the motion-captured and force plate data. The gait cycle was defined as the time interval between two successive right heel-contacts. The marker data were low-pass filtered at 7 Hz using a zero-phase shift lowpass filter^46^. No lowpass filtering was applied to the GRF signals. To quantify the three-dimensional (3D) body kinematics, a segment-fixed coordinate system was defined for each of the 13 body segments (head, thorax, pelvis, right and left upper arms, forearms, thighs, shanks, and feet) using the attached markers (Supplementary Fig. S5). The x-, y-, and z-axes approximately pointed to the anterior, left, and superior directions, respectively. The segmental angles, i.e., the 3D orientations of the body segments with respect to the laboratory coordinate system, were quantified using the y–x–z Euler angles. The joint angles of the hip, knee, and ankle were calculated as the motions of the distal segment coordinate systems with respect to the proximal coordinate systems using the y–x–z Euler angles. The joint angles were set to zero in a quiet standing posture. For comparisons, the GRFs were normalised by the body mass × the gravitational acceleration. The VFM was normalised by the product of the body mass, gravitational acceleration, and COM height^47^.

### Calculation of whole-body angular momentum

A 15-segment whole-body model, consisting of the above 13 segments and hands defined as point masses, was used to calculate the WBAM regarding the body’s COM. The WBAM concerning the body’s COM, **L**, was calculated as the sum of the individual segment angular momenta as in the study by Herr and Popovic^47^:

1 $${\mathbf{L}} = \mathop \sum \limits_{i = 1}^{15} \left( {{\mathbf{c}}_{i} \times m_{i} {\dot{\mathbf{c}}}_{i} + {\mathbf{I}}_{i} {{\varvec{\upomega}}}_{i} } \right),$$

where *m*_*i*_ is the mass, **I**_*i*_ is the inertia tensor of the segment’s COM, **c**_*i*_ is the COM position vector, and ${\dot{\mathbf{c}}}_{i}$ is the COM velocity vector (time derivative of **c**_*i*_), with respect to the whole-body COM, of the *i*-th segment. **ω**_*i*_ is the angular velocity vector of the *i*-th segment, all represented in the laboratory coordinate system. The segmental masses, inertia tensors, and COM positions of each participant were estimated based on the measured marker positions and the anthropometric parameters (the ratio of the segment mass to the total body mass, the segment’s COM location along its longitudinal axis as a percentage of the segment length, and the radii of gyration of the segment as percentages of the segment length) as presented in the studies by de Leva^48^ and Zatsiorsky^49^. The WBAM was normalised by the product of the body mass, COM height, and walking velocity^47^.

### Calculation of net external moment due to GRFs and VFM

The net external moments of the body’s COM due to GRFs and VFMs, **M**, can be calculated as follows:

2 $${\mathbf{M}} = {\mathbf{r}}_{L} \times {\mathbf{F}}_{L} + {\mathbf{r}}_{R} \times {\mathbf{F}}_{R} + {{\varvec{\uptau}}}_{L} + {{\varvec{\uptau}}}_{R} ,$$

where ${\mathbf{r}}_{L}$ and ${\mathbf{r}}_{R}$ are the vectors pointing the left and right centres of pressure, respectively, from the body’s COM, ${\mathbf{F}}_{L}$ and ${\mathbf{F}}_{R}$ are the left and right GRF vectors, respectively, and ${{\varvec{\uptau}}}_{L}={(0, 0, {\tau }_{zL})}^{T}$ and ${{\varvec{\uptau}}}_{R}={(0, 0, {\tau }_{zR})}^{T}$ are the VFM vectors acting on the left and right feet, respectively. The external moments were normalised by the product of the body mass, gravitational acceleration, and COM height^47^.

### Statistical analysis

To test for significant differences in the mean peak values of the measured quantities (i.e. GRF, VFM, segmental angles, joint angles, WBAM, and moments) among three conditions (PCwoAS, NLwoAS, and PCwAS), one-way repeated measures of analysis of variance with post hoc Bonferroni tests were performed. If the normality or homogeneity was violated using the Shapiro–Wilk normality test, Friedman tests and nonparametric Bonferroni tests were used. The statistical significance level was set at *p* < 0.05. All statistical analyses were performed using R, version 4.1.2 (R Foundation for Statistical Computing, Vienna, Austria)^50^.

## Supplementary Information

Supplementary Figures.

## Data Availability

All data are included in the manuscript and/or supporting information.

## Code availability

The computer code used for this study is available from the corresponding author upon reasonable request.
