# Supplementary material for: Functional significance of vertical free moment for generation of human bipedal walking
Source: Sci Rep. 2023 Apr 27;13:6894. doi: 10.1038/s41598-023-34153-4 (PMC10140179; doi:10.1038/s41598-023-34153-4)
Supplement: Supplementary file 1 — Supplementary Figures. [file 41598_2023_34153_MOESM1_ESM.pdf]

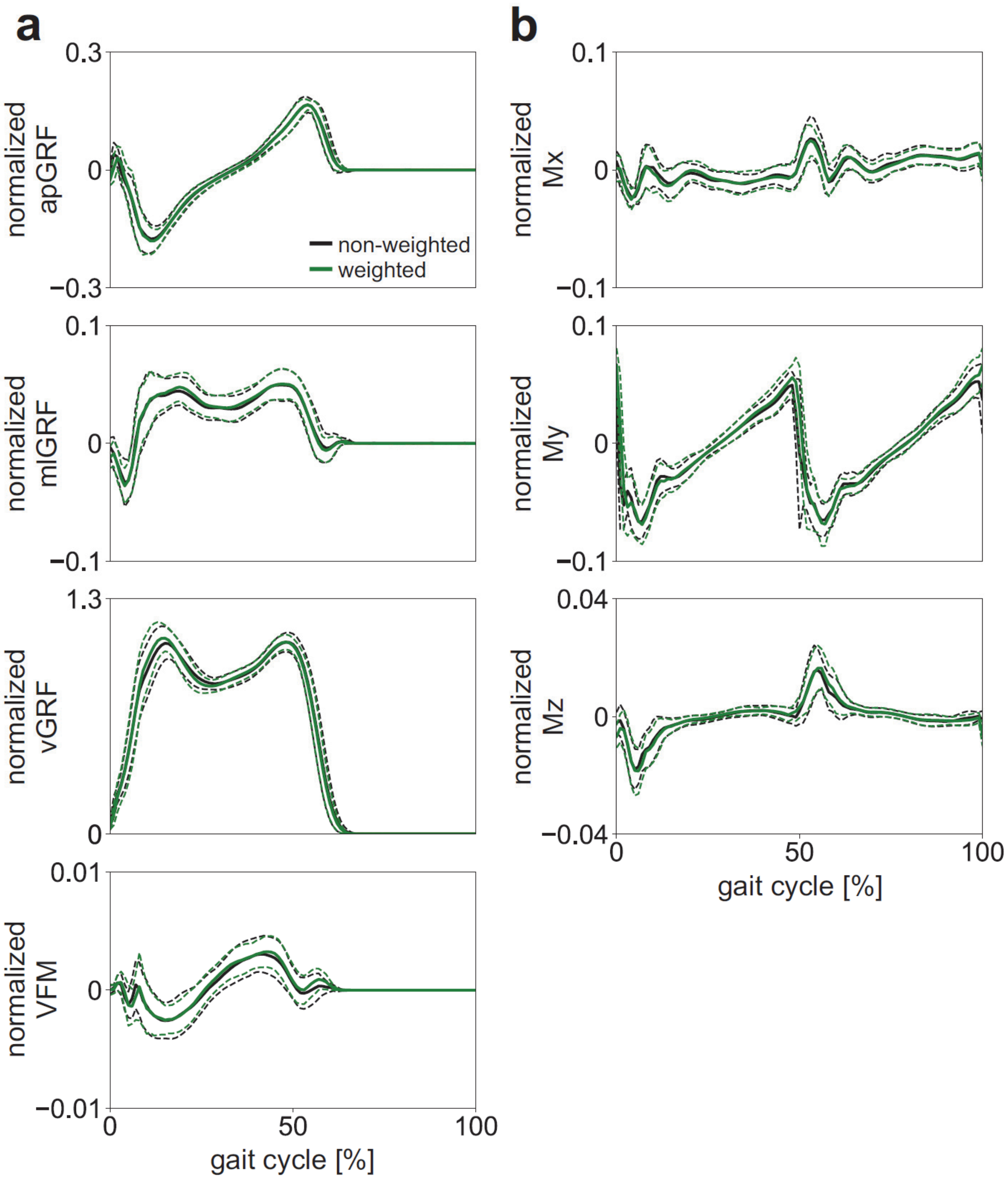

Figure S1. Mean normalized GRF, VFM (**a**) and external moment (**b**) profiles during walking with non-weighted (black) and weighted (green) shoes. Corresponding dashed lines represent standard deviations. Walking with weighted shoes had no effect on the kinetics of walking.

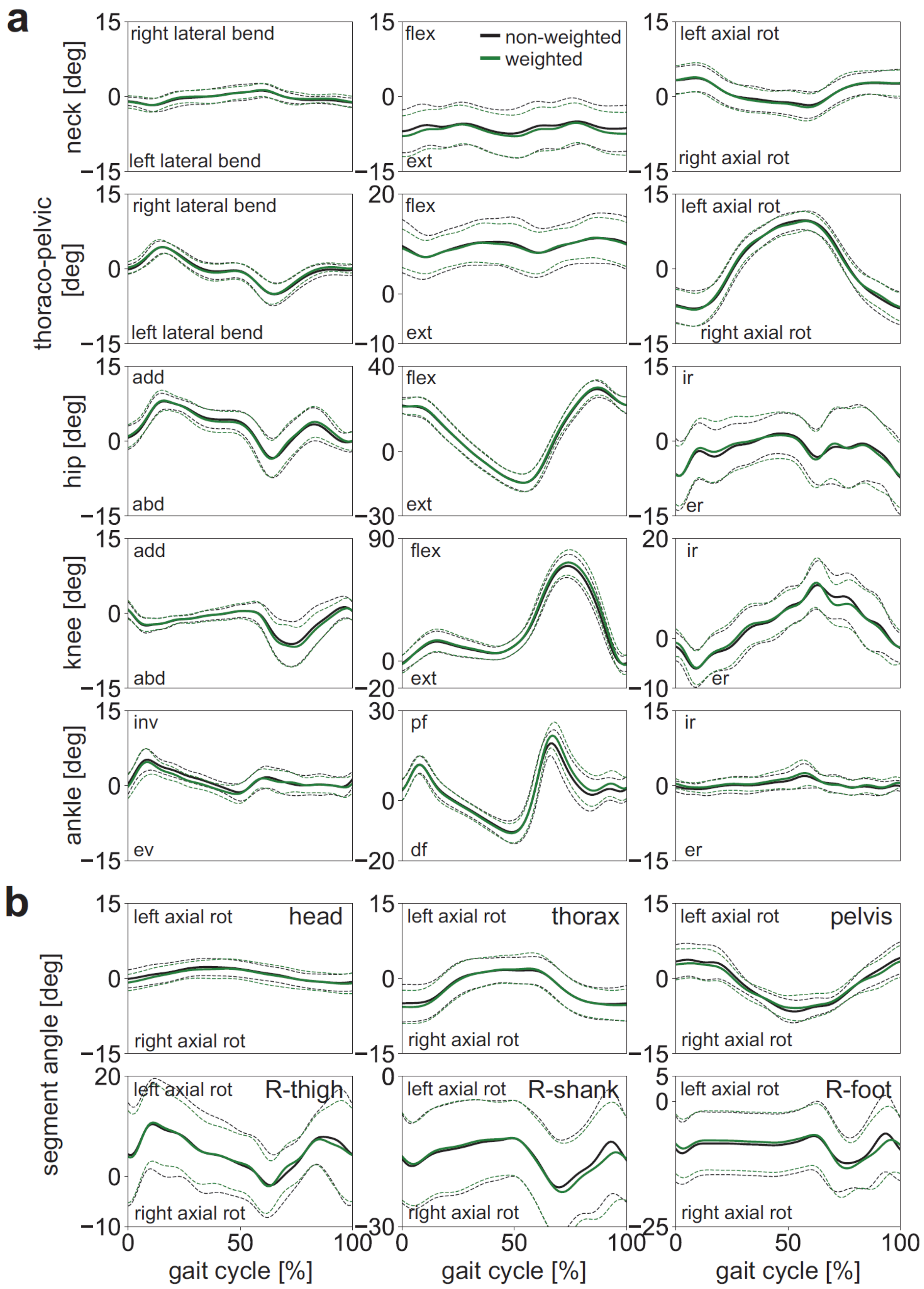

Figure S2. Mean joint (**a**) and segment (**b**) angle profiles during walking with non-weighted (black) and weighted (green) shoes. Corresponding dashed lines represent standard deviations. Walking with weighted shoes had no effect on the kinematics of walking.

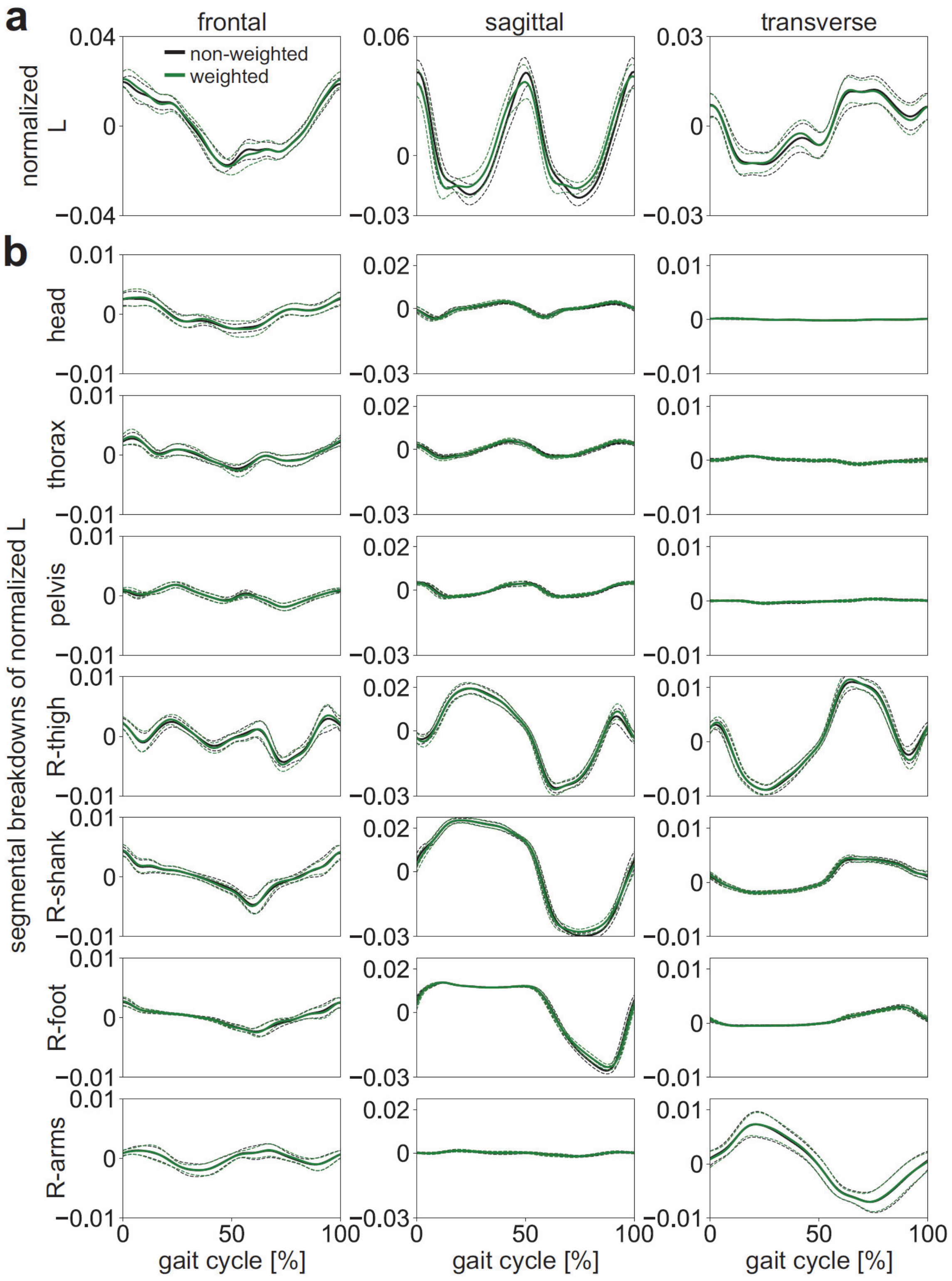

Figure S3. Mean normalized WBAM (**a**) and segmental breakdowns of the mean normalized WBAM (**b**) profiles in the frontal plane (about the anteroposterior axis), sagittal plane (about the mediolateral axis), and horizontal plane (about the vertical axis) during walking with non-weighted (black) and weighted (green) shoes. Corresponding dashed lines represent standard deviations. Walking with the weighted shoes had no effect on the segmental angular momenta during walking.

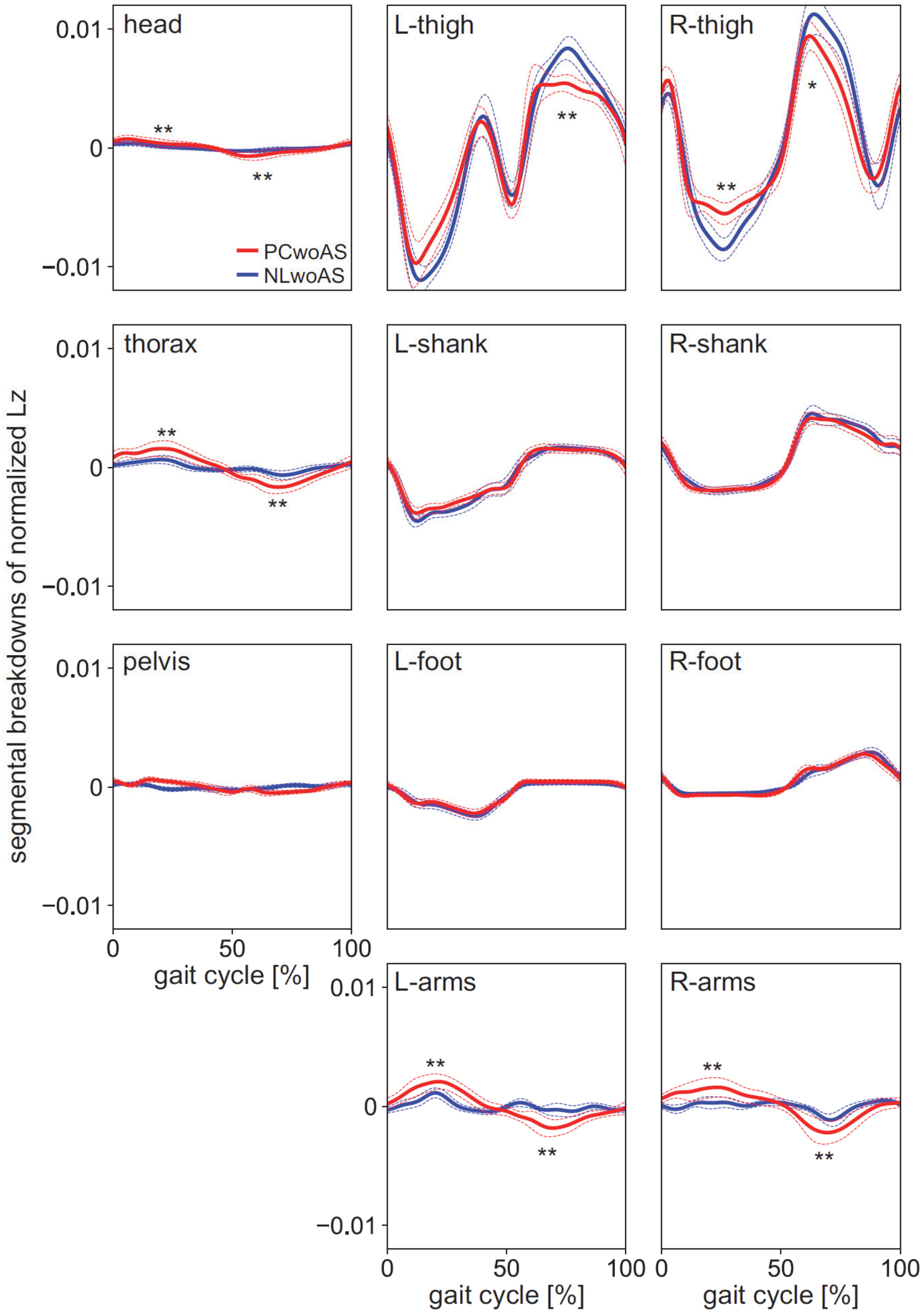

Figure S4. Segmental breakdowns of the mean normalized WBAM in the transverse plane (about the vertical axis) during walking. Angular momenta of the upper arm, forearm, and hand were consolidated for the sake of simplicity. Blue solid line = NLwoAS, Red solid line = PCwoAS. Corresponding dashed lines represent standard deviations. Asterisks indicate statistical differences of the maximum or minimum values (*: $p < 0.05$. **: $p < 0.01$). The amplitudes of the segmental angular momenta of the thigh segments were reduced and those of the thorax and arms were increased in walking with the PC shoes to compensate for the loss of the VFM.

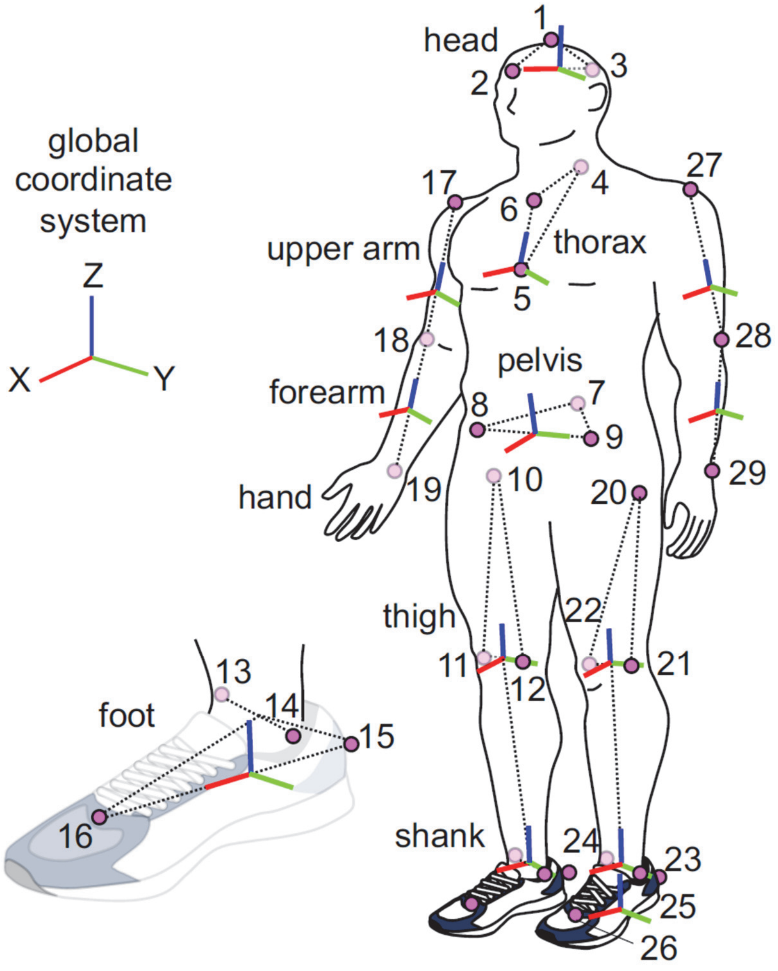

Figure S5. Placement of reflective markers and segment-fixed coordinate systems. A total of 29 markers were attached to (1) top head, (2) front head, (3) rear head, (4) 1st thoracic vertebra, (5) xiphoid process, (6) manubrium of sternum, (7) sacrum, (8,9) anterior superior iliac spine, (10,20) greater trochanter, (11,21) lateral knee, (12,22) medial knee, (13,23) lateral ankle, (14,24) medial ankle, (15,25) heel, (16,26) toe, (17,27) acromion, (18,28) elbow, and (19,29) wrist. Markers on the toes and heels were placed on the corresponding surface positions of the shoes. The hand segment was defined as the point mass in the present study; hence, a segment-fixed coordinate system was not defined for the hand segment.
